# Covid 19, lockdown and brief psychotic disorders

**DOI:** 10.1192/j.eurpsy.2021.1448

**Published:** 2021-08-13

**Authors:** J. Gonçalves Cerejeira, I. Santos Carrasco, C. Capella Meseguer, E. Rodríguez Vázquez, M. Óscar, M. Queipo De Llano, G. Guerra Valera, A. Gonzaga Ramírez

**Affiliations:** 1 Psiquiatría, Hospital Clínico Universitario de Valladolid, Valladolid, Spain; 2 Psiquiatría, Hospital Clínico Universitario de Valladolid, VALLADOLID, Spain; 3 Psichiatry, hospital clinico universitario de Valladolid, VALLADOLID, Spain

**Keywords:** brief psychotic disorder, lockdown

## Abstract

**Introduction:**

Acute and transient psychotic disorders are a rare condition entity as the sudden appearance of affective, confusional symptoms and paranoia triggered by some psychological trauma. The current pandemic caused by COVID-19 is an important psychological stressor that could favor the appearance of acute psychotic disorders. Several studies have been recently published proposing that the multifactorial stress associated with lockdown could function as a catalyst for acute psychotic disorders.

**Objectives:**

To present a case of a brief psychotic disorder during the national lockdown in Spain and to review the literature about the relationship between the current pandemic and psychosis.

**Methods:**

We will present a case report and a literature review.

**Results:**

We report a case of a 27-year-old woman, with no previous psychiatric history. Three weeks after the start of Spain lockdown sudden symptoms appeared with psychomotor restlessness, confused speech, emotional lability, thought blocking and persecutory and referential delusions. Physical exam, blood analysis and cerebral CT scan with no alterations. Treatment was performed with aripiprazole 10 mg and lorazepam 1 mg daily with clinical improvement in one weeks. She was diagnosed of Acute transient psychotic disorder.

**Conclusions:**

Stressful life events that can trigger psychosis in vulnerable individuals and the current pandemic and lockdown context could favor the appearance of acute psychotic disorders. The case reported here is in line with other current studies that show a preliminary intuition of this trend.

